# Antibiotic resistance in *Mycobacterium tuberculosis* alters tolerance to cell wall-targeting inhibitors

**DOI:** 10.1093/jacamr/dlae086

**Published:** 2024-06-04

**Authors:** William J Jowsey, Gregory M Cook, Matthew B McNeil

**Affiliations:** Department of Microbiology and Immunology, University of Otago, Dunedin, New Zealand; Department of Microbiology and Immunology, University of Otago, Dunedin, New Zealand; Department of Microbiology and Immunology, University of Otago, Dunedin, New Zealand

## Abstract

**Background:**

A limited ability to eliminate drug-resistant strains of *Mycobacterium tuberculosis* is a major contributor to the morbidity of TB. Complicating this problem, little is known about how drug resistance-conferring mutations alter the ability of *M. tuberculosis* to tolerate antibiotic killing. Here, we investigated if drug-resistant strains of *M. tuberculosis* have an altered ability to tolerate killing by cell wall-targeting inhibitors.

**Methods:**

Bacterial killing and MIC assays were used to test for antibiotic tolerance and synergy against a panel of drug-resistant *M. tuberculosis* strains.

**Results:**

Our results demonstrate that vancomycin and thioacetazone exhibit increased killing of diverse drug-resistant strains. Mutations in *mmaA4* and *mmpL3* increased vancomycin killing, which was consistent with vancomycin synergizing with thioacetazone and MmpL3-targeting inhibitors. In contrast, mutations in the *mce1* operon conferred tolerance to vancomycin.

**Conclusions:**

Overall, this work demonstrates how drug-resistant strains experience perturbations in cell-wall production that alters their tolerance to killing by cell wall-targeting inhibitors.

## Introduction

*Mycobacterium tuberculosis*, the primary infectious agent of TB, remains a leading cause of infectious disease morbidity and mortality.^1^ The ability of *M. tuberculosis* to become resistant to all available antibiotics, including the newly approved antitubercular drugs bedaquiline and pretomanid,^2,3^ underpins its success as an infectious agent. While resistance is characterized by bacterial growth during antibiotic exposure, antibiotic tolerance is broadly defined as the duration and/or concentration of antibiotic exposure bacteria can survive. Recent reports have highlighted significant variations in antibiotic tolerance amongst *M. tuberculosis* clinical isolates that are otherwise phenotypically susceptible.^4^ Importantly, whilst drug resistance is a known risk factor for treatment failure, the influence of antibiotic tolerance on treatment outcomes is poorly understood.

The cell envelope of *M. tuberculosis* acts as a permeability barrier and is comprised of an inner bi-lipid membrane intercalated with sugar-lipids, a mycolyl-arabinogalactan-peptidoglycan cell wall, outer capsule with surface lipids and sugar polymers, and various proteins throughout.^5,6^ Several important antitubercular drugs target cell-wall biosynthesis, including isoniazid, carbapenems, ethambutol and SQ109.^7^ The mycobacterial cell wall is essential for viability, and dysregulation of its biosynthesis frequently results in altered tolerances to cell wall-targeting antibiotics.^8–11^ For example, dysregulation of cell-wall biosynthesis in *M. tuberculosis* rifampicin-resistant isolates leads to decreased tolerance to vancomycin and pretomanid.^12,13^

Here, we hypothesized that drug-resistance mutations would impact mycobacterial physiology, in turn dysregulating cell-wall biosynthesis and altering tolerance to cell wall-targeting antibiotics. We investigated this hypothesis using bacterial killing assays where we tested a panel of isogenic drug-resistant (DR) strains of *M. tuberculosis* mc^2^6206 for altered tolerance to cell wall-targeting inhibitors, including both traditional (e.g. isoniazid) and non-traditional (e.g. vancomycin) antitubercular agents. We observed that genetically diverse DR strains had reduced tolerance to both vancomycin and thioacetazone. Vancomycin also synergized with various cell-wall biosynthesis inhibitors, which was often reciprocal with drug-resistance mutations that targeted the same processes. Combined, these results highlight how physiological dysregulation in DR strains of *M. tuberculosis* generates differences in tolerance to cell wall-targeting antibiotics. This work also further highlights the need to consider how variations in antibiotic tolerance in clinical isolates effects treatment outcomes.

## Materials and methods

### Bacterial strains and growth conditions

This study was conducted with the avirulent attenuated *M. tuberculosis* strain mc^2^6206 (Δ*leuCD*, Δ*panCD*), and a panel of DR strains derived from mc^2^6206 (Table S1, available as Supplementary data at *JAC-AMR* Online). Strains were grown in 7H9 liquid medium supplemented with OADC [0.005% oleic acid (Sigma, #O1008), 0.5% bovine serum albumin (pH Scientific, #PH100), 0.2% dextrose (Sigma, #G8270), 0.085% catalase (Sigma, #02071)] and 25 µg/mL pantothenic acid (Sigma, #21210), 50 µg/mL leucine (Sigma, #L8000) and 0.05% tyloxapol (Sigma, #T8761) or on 7H11 solid media supplemented with OADC and 50 µg/mL leucine. Strains were maintained as previously stated.^13^ All antibiotics, and antibiotic stock concentrations used in this study are listed in Table S2.

### MIC assays

MIC assays were carried out in 96-well flat bottom plates as previously described,^13^ but with the following modifications. Six-point dilutions were performed across plate columns 3–10, with row A containing solvent only and rows B–H containing compound titrations. For nine-point dilutions, in plate rows B–G, column 11 contained solvent only and columns 2–10 contained compound titrations. For both assay configurations, 150 µL of medium was added to the remaining wells (medium sterility controls). Compounds were 2- or 3-fold serially diluted in the appropriate solvent then added at volumes of 1.5 to 4.5 µL (<3% final solvent). For assays containing additional compounds at subinhibitory concentrations, added compounds were made up in media to 2× their final concentrations and then aliquoted into each well (including solvent-only well; <2% final solvent). Cultures were inoculated to a density of OD_600_ ∼0.005 by combining 75 µL of OD_600_ 0.01 culture with medium and compound/solvent present in wells. Following incubation for 10 days at 37°C, OD_600_ values were determined using a Varioskan Lux microplate reader (Thermo Fisher Scientific).

MIC values were determined as previously described.^13^ Briefly, background absorbance (OD_600_ average of wells containing medium sterility controls) was subtracted from test absorbances, which were then expressed as percentages of the absorbance of the solvent-only well. These values were log-transformed to allow for the MIC to be determined by non-linear regression (curve fit) with the Gompertz equation in GraphPad Prism (V10.0.1).

### Killing assays

Killing assays were performed alongside six-point dilution MIC assays. When preparing the assays, Day 0 cfu/mL were taken for each strain by spot plating 5 µL of OD_600_ ∼0.005 culture on supplemented 7H11 agar, as well as three 10-fold serial dilutions (to 1 × 10^−3^; 15 µL culture transferred to 135 µL of 7H9-OADC). Following OD_600_ reads for the MIC, 75 µL of culture from wells containing compound concentrations where the parent strain showed no turbidity was combined with 75 µL of 7H9-OADC. This was then diluted and plated as described above. This process was repeated for two additional compound concentrations and solvent-only culture wells. Spot plates were incubated at 37°C and counted after 4 weeks.

### Killing kinetics

Cultures were diluted to an OD_600_ of 0.1 and 500 µL was transferred to 9.35–9.4 mL of 7H9-supplemented medium in T25 flasks, making the starting OD_600_ 0.005 in a final volume of 10 mL. Following this, 100–150 µL of pre-diluted compound or solvent only was added (1%–1.5% final solvent) and resulting cultures incubated at 37°C. Day 0 cfu/mL were taken for each strain by spot plating 5 µL of culture (after 1/20 dilution of OD_600_ 0.1 culture) as stated above. At Days 3, 5, 7, 10, 14 and 21, 150 µL of culture was removed from each flask and diluted to 1 × 10^−7^ (15 µL of culture transferred to 135 µL of 7H9-OADC), then 5 µL of each dilution was plated on supplemented 7H11 agar. Spot plates were incubated at 37°C and cfu counted as described above.

### Resistant mutant isolation and WGS

Following killing kinetics assays, single colonies were isolated from Day 21 agar plates and passaged at 37°C in 10 mL of supplemented 7H9 medium in T25 flasks. Once cultures appeared turbid, a loopful of culture was spread on supplemented 7H11 agar, from which single colonies were re-isolated and passaged once more. Genomic DNA was extracted using the ZymoBIOMICS DNA miniprep kit (Zymo, #D4300) following the manufacturer’s instructions. Briefly, 5–10 mL of turbid culture (OD_600_ 0.5–1) was centrifuged in 15 mL canonical tubes at 4000 ×**g** for 10 min at 20°C, and cells lysed using a MiniG 1600 tissue homogenizer (SPEX, Metuchen, NJ, USA) at 1500 rpm for 5 min. Following a series of wash steps, DNA was eluted in 50 µL of Milli-Q water heated to 60°C after incubation for 5 min.

DNA preparations were whole-genome sequenced at SeqCenter using their ‘400 Mbp (2.67M Reads)’ Illumina paired-end reads service. Paired-end reads were filtered for 31-mer matches to PhiX (Illumina spike-in) using the BBTools BBDUK script (ref = phix, k = 31, hdist = 1) then run through error correction using the BBTools tadpole script (mode = correct, k = 50). Reads were then aligned to the *M. tuberculosis* H37Rv genome (NC_000962) using Bowtie 2 then mapped and sorted using SAMtools view and sort commands. Variants were called using BCFtools mpileup command followed by call with -m and -v parameters. After filtering the variants for a quality score of >50, the effect of the variants was estimated using the SnpEff eff command, the output from which was manually inspected. Variants in PE/PPE genes and with more than one genotype were disregarded. Unique variants (i.e. between mc^2^6206 strains) were manually inspected using the Integrative Genomics Viewer web application (https://igv.org/app/) with the *M. tuberculosis* H37Rv genome and annotations. Variants are presented in Table S1. BAM files from WGS of vancomycin-tolerant isolates can be accessed via NCBI under the BioProject accession PRJNA1106920.

## Results

### DR strains of M. tuberculosis show diverse tolerances to cell wall-targeting antibiotics

**Figure 1.**
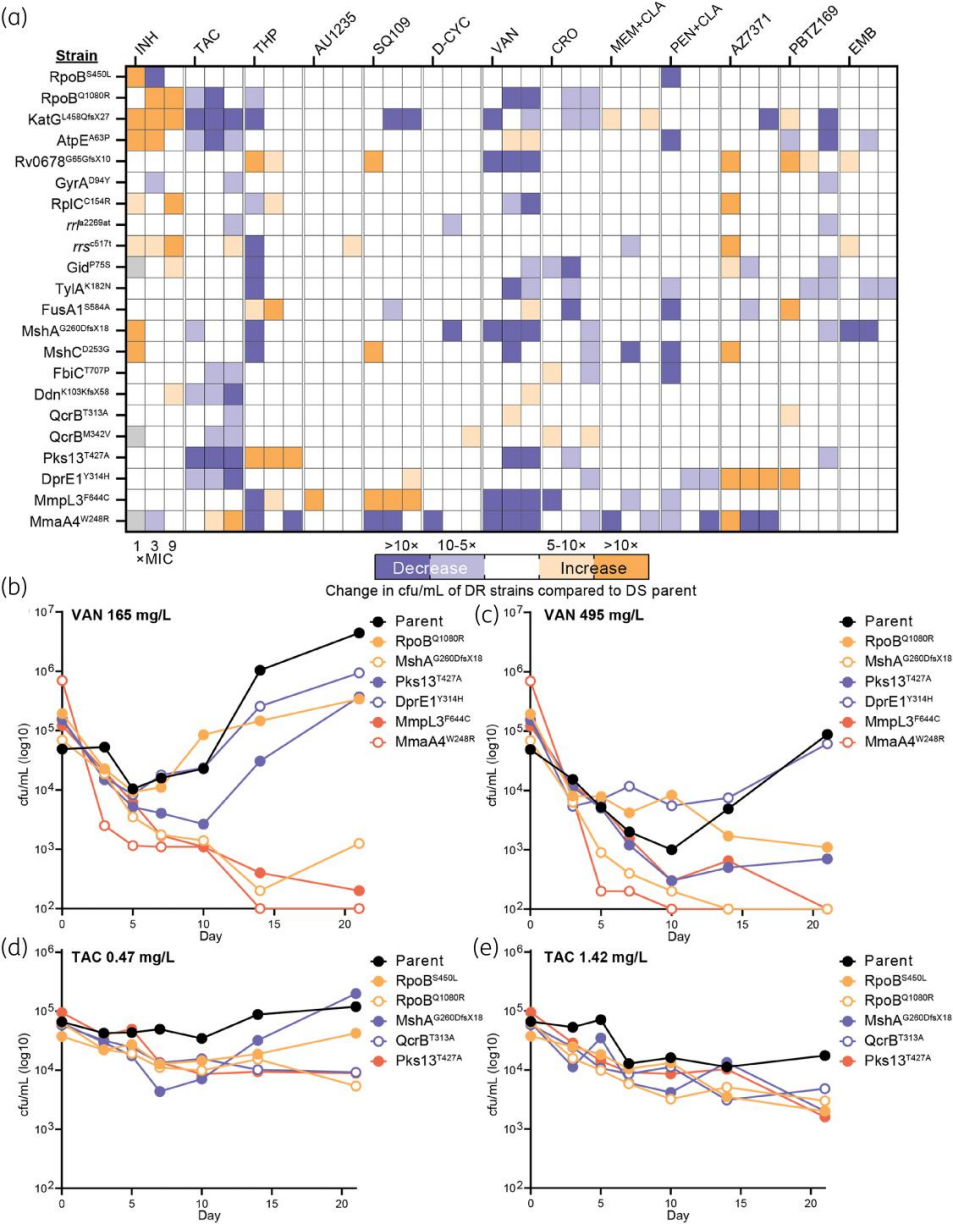
Comparisons of antibiotic killing against the DS parent and DR strains of *M. tuberculosis*. (a) Heatmap of killing assays showing changes in cfu/mL of DR strains compared with the parent. See key for colour coding. Grey boxes indicate no data for the given test. For each antibiotic tested (denoted at top), cultures from three concentrations corresponding to 1 (little-to-no turbidity), 3 and 9× the DS parent MIC were spot plated. For vancomycin and D-CYC, concentrations of 0.33, 1 and 3× the parent MIC were plated. 1× MICs (mg/L, rounded to two decimal places except PBTZ169): INH, isoniazid, 0.07; TAC, thioacetazone, 0.47; THP, thiophene-2, 1.26; AU1235, 0.1; SQ109, 0.2; D-CYC, D-cycloserine, 27.56; VAN, vancomycin, 165; CRO, ceftriaxone, 52.93; MEM, meropenem, 2.63; PEN, penicillin, 14.97; AZ7371, 1.07; PBTZ169, 0.0007; EMB, ethambutol, 2.77. Clavulanic acid (where denoted by +CLA) was added to 47.45 mg/L. cfu/mL were calculated from the average from two replicates (except vancomycin and thioacetazone, *n* = 4). Relative values were calculated by dividing Day10/Day0 cfu/mL of DR strains by Day10/Day0 cfu/mL of the DS parent at each antibiotic concentration. (b, c) Killing kinetics of a selection of DR strains and the DS parent in vancomycin challenges at 1 and 3× the DS parent MIC (165 and 495 mg/L, respectively). Circles denote mean cfu/mL values from biological duplicate assays. Lower limit of detection was 200 cfu/mL. (d, e) Same as (b, c) but with thioacetazone (at 0.47 and 1.42 mg/L, respectively).

We hypothesized that DR strains of *M. tuberculosis* would be dysregulated for cell-wall biosynthesis and have altered tolerance to cell wall-targeting antibiotics. To investigate this, we tested a panel of 22 DR strains against 13 cell wall-targeting antibiotics for increased killing compared with the drug susceptible (DS) parent strain (mc^2^6206). This screen identified known resistance phenotypes including resistance of (i) mutant *katG* and *mshA* against isoniazid, (ii) mutant Rv0678 against various antibiotics, (iii) mutant *mmpL3* to AU1235 and SQ109, and (iv) mutant *dprE1* against AZ7371 and PBTZ169 (Figure 1a).^13^ Multiple DR strains showed reduced viability compared with the DS parent when challenged with either vancomycin or thioacetazone (Figure 1a). This included strains with mutations in cell-wall biosynthesis genes (e.g. *mmpL3*, *pks13*, *mmaA4*, *dprE1*), central cellular pathways (e.g. *rplC*, *rpoB*), and in the detoxification of oxidative stress (e.g. *katG*, *mshA*). Similar broad decreases were observed with thiophene-2, but only at the DS parent MIC (Figure 1a). Strains with mutations in respiratory and ATP synthesis genes (e.g. *ddn*, *qcrB*, *atpE*) showed increased tolerance against vancomycin (Figure 1a). Ceftriaxone, penicillin, meropenem and D-cycloserine, despite all being inhibitors of peptidoglycan biosynthesis like vancomycin, had very little overlap with vancomycin in increased killing of DR strains (Figure 1a). This possibly reflects differences in the cell-wall permeability of these molecules, or their molecular targets as only vancomycin directly binds peptidoglycan.

We next carried out time–kill assays to validate the increased lethality of vancomycin and thioacetazone. When used at 1× the parent MIC (165 mg/L), vancomycin was bacteriostatic against the DS parent but bactericidal against DR strains MmaA4^W248R^, MmpL3^F644C^ and MshA^G260DfsX18^ (Figure 1b). Cultures of all strains, apart from MmaA4^W248R^, MmpL3^F644C^ and MshA^G260DfsX18^, increased in cfu/mL after Day 10, suggestive of phenotypic or genetic adaptations to vancomycin. All strains showed increased rates of killing when challenged with 3 ×  vancomycin (495 mg/L), yet only WT and DprE1^Y314H^ showed a recovery in cfu/mL after Day 10 (Figure 1c). In contrast to vancomycin when used at parental MIC (165 mg/L), RpoB^Q1080R^ and Pks13^T427A^ showed no recovery in cfu/ml, MmpL3^F644C^ and MshAG^260DfsX18^ cfu/mL were reduced to below the level of detection, and the cfu/mL of MmaA4^W248R^ was reduced (>3 log_10_) by Day 5 (Figure 1c). Whilst DR strains showed reduced tolerance to thioacetazone in killing assays, only RpoB^Q1080R^ and Pks13^T427A^ had 1 log_10_ reductions in viability at 1× the parent MIC (0.47 mg/L) of thioacetazone by Day 21 in time–kill assays. By Day 21 of the 3× parent MIC (1.42 mg/L) thioacetazone challenge, the DS parent had a ∼0.5 log_10_ reduction, while many of the DR strains showed >1 log_10_ reductions in viability (Figure 1b). In summary, a broad range of DR strains exhibited reduced tolerance to vancomycin and, to a lesser extent, thioacetazone.

### mmpL3 and mmaA4 mutations, and not phthiocerol dimycocerosate biosynthesis gene mutations, drive vancomycin killing

**Figure 2.**
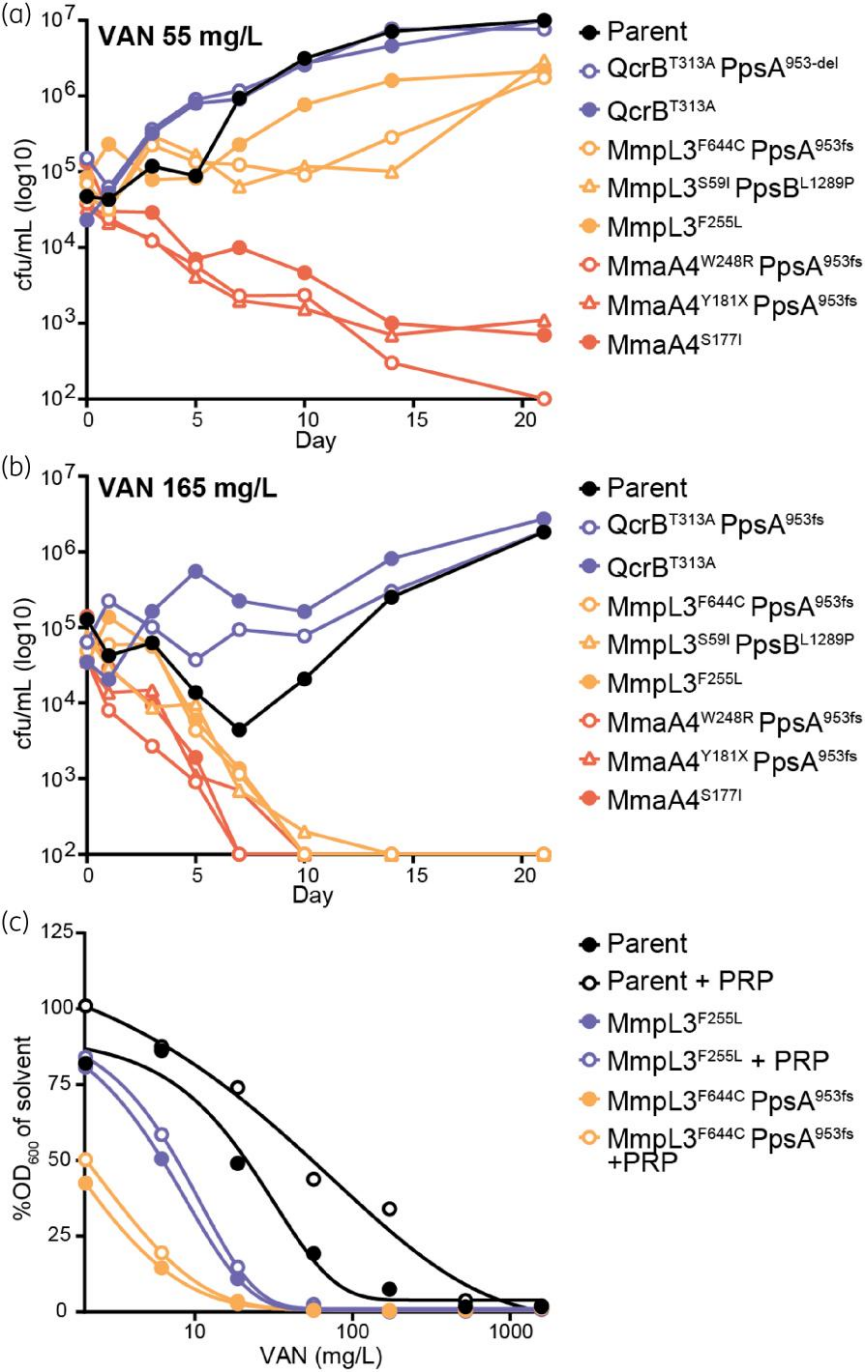
Comparisons of DR strains harbouring WT or mutant PDIM biosynthesis genes when challenged with vancomycin. (a, b) Killing kinetics of DR strains with 0.33 (55 mg/L) and 1× (165 mg/L) the vancomycin MIC of the DS parent, respectively. Symbols show mean cfu/mL values from biological duplicate assays. Closed symbols indicate strains with WT PDIM genes; open symbols indicate mutant PDIM genes (denoted in key). Lower limit of detection was 200 cfu/mL. (c) Vancomycin MIC assay with or without 0.1 mM supplementation of propionate. Circles show mean OD_600_ values from biological duplicate assays (each with two technical replicates). Open circles indicate propionate supplementation (denoted by +PRP), and closed circles solvent only. PpsA^T953TfsX120^ is denoted as PpsA^953fs^. Raw OD_600_ values, including those of MmaA4^181-del^ and MmaA4^S177I^ are presented in Figure S1.

Phthiocerol dimycocerosates (PDIM) are lipids that populate the outer layer of the *M. tuberculosis* cell envelope, and loss of PDIM biosynthesis increases vancomycin susceptibility.^14–17^ Vancomycin exhibited increased killing of multiple DR strains of *M. tuberculosis* (Figure 1), yet some strains including MmpL3^F644C^ and MmaA4^W248R^ contained mutations in PDIM biosynthesis genes (e.g. *ppsA*, *ppsB*, *ppsC* and *fadD26*; Table S1). Despite this, we reasoned mutant *mmpL3* and *mmaA4*, and not mutated PDIM biosynthesis genes, were primary drivers of vancomycin susceptibility in MmpL3^F644C^ and MmaA4^W248R^. To test this, we first carried out time–kill assays of mutant *mmpL3* and *mmaA4* strains containing either mutant or WT PDIM genes against vancomycin. The *mmaA4* mutants showed comparable rates of killing at 0.33 and 1× the DS parent MIC, irrespective of PDIM gene mutations (Figure 2a, b). All *mmpL3* mutants showed comparable rates of killing at 1× the DS parent vancomycin MIC, although the MmpL3^F265L^ mutant with WT PDIM genes showed improved growth at the vancomycin concentration of 0.33× DS parent MIC (Figure 2a and b). Importantly, there was also no variation between *qcrB* mutants despite one of these strains encoding PpsA^T953TfsX120^ that is also present in strains MmpL3^F644C^ and MmaA4^W248R^ (Figure 2a and b).

Propionate supplementation has recently been reported to increase PDIM biosynthesis in *M. tuberculosis* strains.^17^ To determine if propionate rescued mutant *mmaA4* and *mmpL3* strains with WT PDIM genes we carried out MIC assays in the presence and absence of 0.1 mM propionate. Whilst propionate protected the DS parent against vancomycin, protection did not extend to the DR strains, irrespective of PDIM gene mutations (Figure 2c, Figure S1). In conclusion, DR-conferring mutations in *mmpL3* and *mmaA4*, rather than mutations in the PDIM biosynthesis pathway, were primary drivers in vancomycin susceptibility of strains MmpL3^F644C^ and MmaA4^W248R^.

### Vancomycin synergizes with genetic and chemical disruption of cell-wall biosynthesis pathways

**Figure 3.**
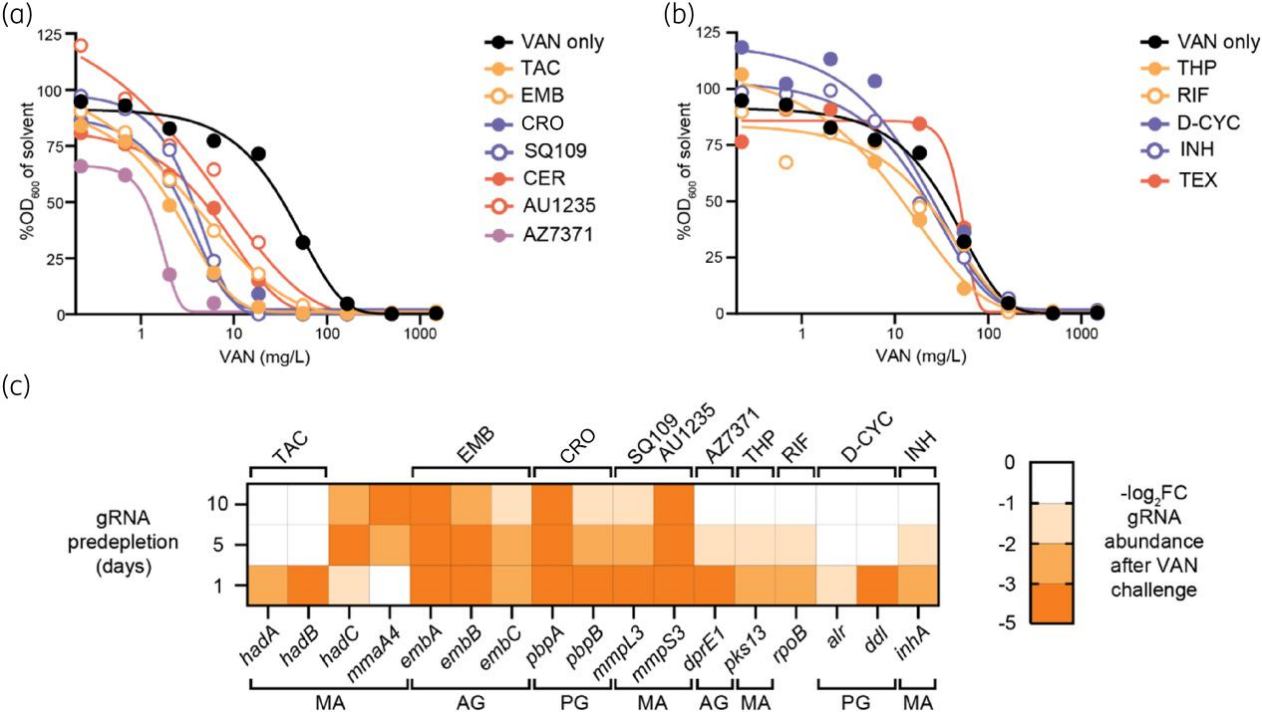
Disruption of cell-wall biosynthesis increases vancomycin susceptibility of *M. tuberculosis*. Vancomycin interactions with cell-wall inhibitors against the DS parent and vancomycin-gene interactions informed from CRISPRi.^19^ (a, b) MIC curves from vancomycin challenge (0.22–1484 mg/L) against the DS parent in combination with subinhibitory concentrations of synergizing and non-synergizing drugs, respectively. Drug concentrations (mg/L, rounded to two decimal places): TAC, thioacetazone, 0.05; EMB, ethambutol, 0.32; CRO, ceftriaxone, 8.27; SQ109, 0.07; CER, cerulenin, 0.18; AU1235, 0.02; AZ7371, 0.18; THP, thiophene-2, 0.14; RIF, rifampicin, 0.05; D-CYC, D-cycloserine, 5.1; INH, isoniazid, 0.02; TEX, teixobactin, 5.8. Dose response curves show mean %OD_600_ values (treatment/solvent OD_600_ × 100) from biological duplicate assays (each with two technical replicates). OD_600_ values are presented in Figure S2. (c) Heatmap of vancomycin-gene interactions from CRISPRi studies,^19^ with target gene gRNA depletion of log_2_ fold change (denoted FC) of −1 or less. Genes are denoted below, with respective cell-wall biosynthesis pathways at the bottom (MA, mycolic acids; PG, peptidoglycan; AG, arabinogalactan). Antibiotics relevant to the genes’ functions are denoted on top.

To further explore which cell-wall biosynthesis processes, when inhibited, improved vancomycin efficacy, we performed vancomycin MIC assays in the presence of subinhibitory concentrations of various cell wall-targeting antibiotics (∼0.11 or 0.33× DS parent MIC). Consistent with previous reports, vancomycin synergized with ethambutol and cerulenin (Figure 3a).^8,16,18^ Vancomycin also synergized with thioacetazone, ceftriaxone, SQ109, AU1235 and AZ7371, with subinhibitory concentrations of each antibiotic decreasing the vancomycin MIC by more than 2-fold (Figure 3a, Table S3). Thioacetazone caused significant growth inhibition at ∼0.33× MIC (0.14 mg/L), yet even when used at ∼0.11× MIC (0.05 mg/L) thioacetazone reduced the vancomycin MIC by more than 10-fold (Figure S2). Isoniazid, D-cycloserine and teixobactin showed no interaction with vancomycin (Figure 3b).

We next hypothesized that vancomycin-susceptible DR strains would become hypersusceptible to vancomycin when challenged against vancomycin in combination with subinhibitory concentrations of vancomycin-synergizing antibiotics (Figure 3a). To test this, we determined vancomycin MICs of the MmaA4^S177I^ and MmpL3^F255L^ strains (containing WT PDIM genes) in the presence of subinhibitory concentrations of vancomycin-synergizing cell wall-targeting antibiotics. Consistent with MmaA4^S177I^ being thioacetazone resistant, subinhibitory concentrations of thioacetazone did not reduce the vancomycin MIC of MmaA4^S177I^ (Table 1). Similarly, the MmpL3-targeting compound SQ109 only partially reduced the vancomycin MIC of MmpL3^F255L^ that is resistant to SQ109 (Table 1, Figure S3). Aside from thioacetazone and SQ109, subinhibitory concentrations of most drugs tested produced synergy-like reductions in the vancomycin MICs of MmaA4^S177I^ and MmpL3^F255L^ (Table 1, Figure S4). However, fold-change reductions in vancomycin MICs elicited by these drugs were weaker for MmaA4^S177I^ and MmpL3^F255L^ than for the DS parent (Table 1). For example, AZ7371 (at 0.18 mg/L) reduced the vancomycin MIC in the parent strain by >50×, whereas AZ7371 reduced the vancomycin MIC of both MmaA4^S177I^ and MmpL3^F255L^ strains by <10× (Table 1). In particular, MmaA4^S177I^ showed smaller reductions in its vancomycin MIC when challenged against vancomycin with vancomycin-synergizing antibiotics (Table 1).

**Table 1.** Vancomycin (VAN)-interacting inhibitors synergize with vancomycin against DR strains

Drugs		VAN MIC (mg/L)^a^			Fold reduction (×MIC)^b^		
Drug	×MIC parent^c^	Parent	MmpL3^F255L^	MmaA4^S177I^	Parent	MmpL3^F255L^	MmaA4^S177I^
VAN	—	148.44	18.50	1.95	1.00	1.00	1.00
TAC	0.11	10.25	2.31	4.22	14.48	8.01	0.46
EMB	0.33	37.02	5.13	0.38	4.01	3.61	5.19
CRO	0.11	106.58	8.13	1.01	1.39	2.27	1.94
CER	0.11	77.52	6.41	0.43	1.91	2.88	4.51
SQ109	0.33	9.64	7.75	0.61	15.40	2.39	3.20
INH	0.33	100.80	7.92	1.87	1.47	2.34	1.04
AZ7371	0.33	2.62	4.99	0.23	56.73	3.71	8.75

Drug concentrations (mg/L, rounded to two decimal places): TAC, thioacetazone, 0.05; EMB, ethambutol, 0.32; CRO, ceftriaxone, 2.75; CER, cerulenin, 0.06; SQ109, 0.07; INH, isoniazid, 0.02; AZ7371, 0.18. OD_600_ data for all drug challenges are presented in Figure S4.

^a^VAN MIC values were calculated from two biological replicate assays using non-linear regression (curve fit) with the Gompertz equation (rounded to two decimal places) (except MmaA4^S177I^* + *AZ7371, which was the lowest vancomycin concentration without growth among replicates; 0.23 mg/L; see Figure S4).

^b^Fold reduction was calculated for each strain by dividing each of their vancomycin-only MICs by vancomycin–drug MICs (before rounding).

^c^*^^* × MIC values were calculated from representative biological assays (each with two technical replicates) as described above [data generated during killing assays (Figure 1a); MIC data not shown].

To further investigate interactions between cell-wall disruption and vancomycin, we surveyed a prior CRISPRi chemical-genetic study for altered vancomycin susceptibility.^19^ Briefly, genes involved in either mycolic acid, peptidoglycan or arabinogalactan synthesis were inspected for guide RNA (gRNA) depletion (−log_2_ fold change) in the high vancomycin treatment. Consistent with the drugs that synergized with vancomycin, gRNAs targeting genes involved in mycolic acid synthesis and polymerization of peptidoglycan and arabinogalactan were more often depleted following vancomycin challenge (Figure 3c). Furthermore, gRNAs targeting *mmpS3*, *mmpL3* and *mmaA4* were depleted, supporting our observations that DR strains encoding mutant MmpL3 and MmaA4 have increased vancomycin susceptibility (Figure 3c). gRNAs targeting *mmaA4* were most depleted after a 10 day gRNA predepletion, possibly reflecting the time needed to induce changes in mycolic acid composition. Interestingly, gRNAs targeting either *hadA* or *hadB*, which produce the HadAB complex targeted by thioacetazone,^20^ were only depleted after 1 day of gRNA predepletion, while *hadC*, which forms a complex with *hadB*, was depleted after each timepoint. The gRNAs targeting genes that had no interaction with vancomycin (i.e. isoniazid/*inhA*, thiophene-2/*pks13*, D-cycloserine/*alr* and *ddl*) showed a relatively small level of depletion following vancomycin challenge and were observed only after the 1 and 5 day gRNA predepletions (Figure 3c). In conclusion, specific chemical and genetic disruptions to cell-wall biosynthesis produce synergistic interactions with vancomycin.

### Tolerance to vancomycin can be acquired through mutation of mce1 operon genes

**Figure 4.**
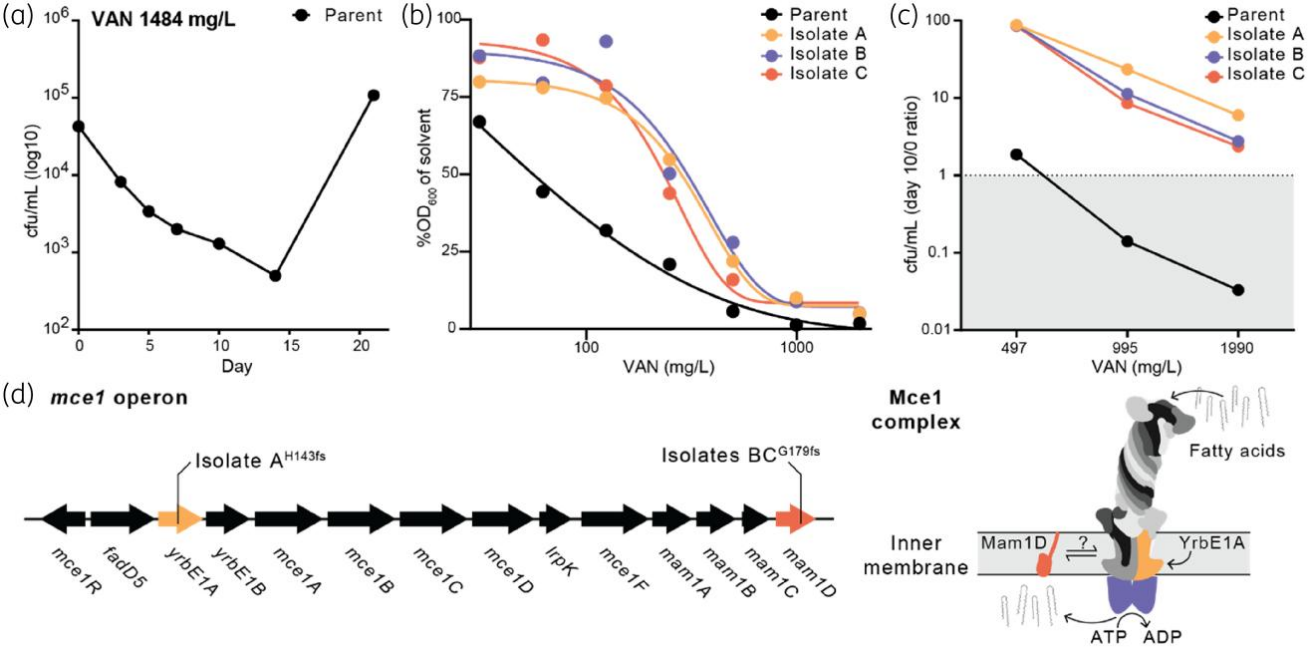
Isolation and characterization of vancomycin (VAN)-tolerant *M. tuberculosis* strains. (a) Killing kinetics of the DS parent at 9× (1484 mg/L) the MIC of vancomycin. Circles show mean cfu/mL values from biological duplicate assays. Lower limit of detection was 200 cfu/mL. (b) MIC curves of vancomycin treatment (31–1990 mg/L) against the DS parent and vancomycin-tolerant isolates. Circles show mean %OD_600_ values (treatment/solvent OD_600_ × 100) from a representative biological assay (*n* = 2, each with two technical replicates). (c) Killing assays plated from (b), with values expressing the relative cfu/mL of treatment (Day 10) versus inoculum (Day 0). The dotted black line indicates inoculum cfu/mL. (d) Representation of the *mce1* operon and Mce1 protein complex with associated vancomycin tolerance-conferring mutations from vancomycin-tolerant isolates annotated. Blue shapes represent Mce1G, which is encoded outside of the *mce1* operon. The ‘?’ indicates that how Mam1D and Mce1 interact is unknown.

In time–kill assays challenging the DS parent with vancomycin at 9 ×  MIC (1484 mg/L), we observed a bactericidal effect with a >1 log_10_ reduction in viability over the first 14 days followed by a sharp increase in cfu/mL (Figure 4a). This inflection was consistent with the emergence of resistant cells following exposure to a bactericidal antibiotic, like that seen for isoniazid.^21^ To determine if these cultures were indeed made up of resistant clones, we isolated single colonies from Day 21 (denoted A, B, C) and tested each for altered vancomycin susceptibility. All isolates had a ∼2× increase in vancomycin MIC compared with the DS parent, and in killing assays showed improved tolerance, with a >1 log_10_ increase in viability at tested concentrations (Figure 4b and c). WGS of each isolate identified non-synonymous mutations in the *mce1* operon that produces an ATP-dependent importer of fatty acids.^22–25^ Isolate A contained a 7 bp insertion that resulted in a frameshift following His143 of *yrbE1A* (Rv0167), whilst isolates B and C contained the same 1 bp deletion that caused a frameshift at Gly179 in the putative upstream accessory protein *mam1D* (Rv0178) (Figure 4d). Consistent with *yrbE1A* and *mam1D* mutations providing vancomycin tolerance-like phenotypes, gRNAs of neither gene were depleted following vancomycin challenges in CRISPRi; *mceD1* was the only *mce1* operon gene with gRNA fold changes below −1 log_2_ (in low and medium vancomycin challenges only).^19^ Overall, mutations in the fatty acid importer genes of the *mce1* operon that likely impair fatty acid import confer vancomycin tolerance-like phenotypes in *M. tuberculosis*.

## Discussion

This study demonstrates how mutations that confer drug resistance in *M. tuberculosis* can reduce tolerance to cell wall-targeting inhibitors that target specific aspects of cell-wall biosynthesis. The most vancomycin-susceptible DR strains contained mutations in *mmpL3* or *mmaA4* genes, which are involved in mycolic acid production, whilst mutations in the *mce1* operon, which encodes a fatty acid importer, were associated with increased vancomycin tolerance. Furthermore, chemical inhibitors that affected the same processes as the mutations in vancomycin-susceptible strains were synergistic with vancomycin. This work highlights how DR strains of *M. tuberculosis* are differentially tolerant to antibiotic killing and how combinations of cell-wall inhibitors can interact synergistically.

**Figure 5.**
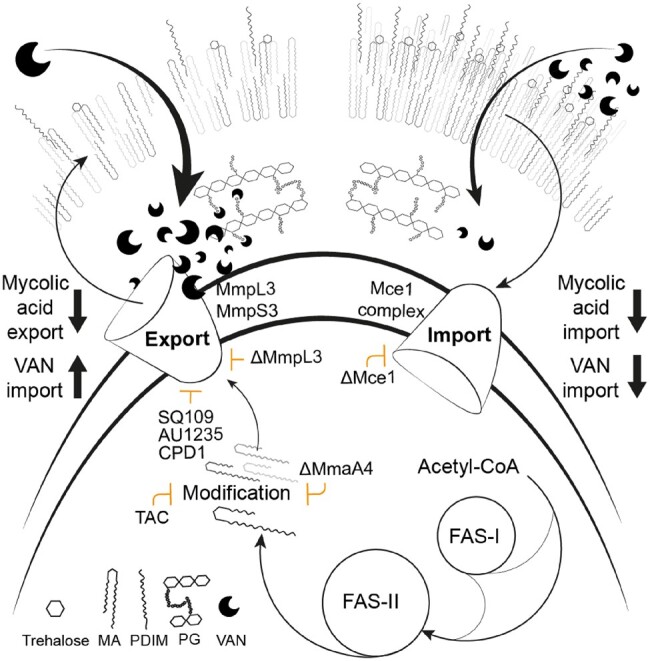
Model of vancomycin activity as a function of mycolic acid layer integrity. Outline of mycolic acid synthesis with processes found to synergize with vancomycin when dysregulated by key antibiotics and DR-conferring mutations depicted. Orange lines indicate the processes effected by antibiotics and mutations. PG, peptidoglycan; MA, mycolic acid; FAS, fatty acid biosynthesis. Mycolic acid species are represented by different shades of grey. The curved double black line represents the inner membrane.

Irregular mycolic acid composition and concentration potentiate vancomycin susceptibility in *M. tuberculosis*. Strains with mutations in mycolic acid biosynthesis genes (e.g. *mmpL3*, *mmaA4*, *pks13*) and chemical inhibitors of mycolic acid biosynthesis (e.g. thioacetazone, SQ109) increased the activity of vancomycin. *mmaA4* encodes a mycolic acid methyltransferase, with its deletion resulting in depletion of methoxy- and keto-mycolic acids,^26^ whilst TAC treatment reduces mycolic acid content.^27,28^ Similarly, inhibition or mutation of *mmpL3*, which encodes the large subunit of the mycolic acid flippase,^29,30^ leads to accumulation of trehalose monomycolates and reduction in mycolic acids and trehalose dimycolates.^31–33^ Together, the reciprocal gene–drug interactions indicate that irregular compositions of cell-wall mycolic acids potentiate vancomycin killing in *M. tuberculosis* (Figure 5).

Cell envelope permeability is a known determinant of vancomycin sensitivity in *M. tuberculosis*, as described for decreased PDIM content.^12,14–16,34,35^ The addition of propionate was unable to restore vancomycin tolerance to mutant *mmpL3* or *mma4* strains possessing WT PDIM genes, demonstrating that vancomycin susceptibility of these strains was mostly a function of altered mycolic acid production and transport. Moreover, cell wall-targeting antibiotics that synergized with vancomycin against the DS parent showed weaker synergy with mutant *mmpL3* or *mma4* strains, suggesting these strains produce cell walls that are likely to be more permeable to vancomycin. As PDIMs and other lipid species are inserted into the mycolic acid layer of the cell wall, these data suggest perturbed mycolic acid production limits the ability of outer membrane lipids to provide an added layer of protection against vancomycin (Figure 5).

It was unclear why isoniazid showed no interaction with vancomycin despite vancomycin interacting with several mutants and inhibitors of the mycolic acid biosynthesis pathway. Prior CRISPRi studies support this as the predepletion of *inhA* only caused a moderate increase in vancomycin susceptibility.^19^ InhA inhibition by isoniazid halts mycolic acid production,^36,37^ but leads to increased expression of fatty acid synthesis II genes.^38^ We hypothesize that subinhibitory concentrations of isoniazid briefly decreased mycolic acid production, but increased expression of fatty acid synthesis II genes restored production of mycolic acids and negated potential synergistic interactions between isoniazid and vancomycin.

Mutations in two separate genes of the *mce1* operon conferred vancomycin tolerance. The Mce1 complex imports mycolic acids and Mce1 deletion results in the remodelling of cell-wall lipids, accumulation of mycolic acids, and increased expression of fatty acid synthase II genes.^23,24,39^ These observations suggest that increased production and/or decreased recycling of mycolic acids caused by mutant Mce1 complexes thickens the cell wall to provide vancomycin tolerance, a similar strategy to that used by vancomycin-resistant *Staphylococcus aureus*.^40^ Clinical isolates of *M. tuberculosis* commonly contain non-synonymous mutations in the *mce1* operon, with a genetic analysis of 100 clinical isolates identifying 26% of strains having *yreB1A* polymorphisms,^41^ and mutations in *mce1A* and *mce1R* being associated with hypervirulent phenotypes.^42^ Moreover, DR *M. tuberculosis* isolates frequently have thicker cell walls than DS isolates.^43^ We hypothesize that *mce1* gene mutations decrease vancomycin permeability through thickening of the cell wall, in turn providing vancomycin tolerance (Figure 5). Given that non-synonymous *mce1* operon mutations abide in DR clinical isolates, these mutations could be providing a crucial survival advantage and act as precursors to drug resistance for *M. tuberculosis*.

This study exemplifies how physiological variations in DR strains of *M. tuberculosis* can result in altered tolerance to cell wall-targeting antibiotics. We found that *M. tuberculosis* mutations in genes involved in mycolic acid biosynthesis and export, and drugs targeting these same processes interacted synergistically with vancomycin. In contrast, mutations in genes that code for fatty acid import increased vancomycin tolerance. Whilst vancomycin has limited therapeutic applications for TB in clinical settings, this study highlights the potential of chemically optimized glycopeptides in combating drug-resistant strains of *M. tuberculosis* as recently explored with arginine-conjugated vancomycin.^18^ Whilst we have previously shown that thioacetazone can target the collateral sensitivities of isoniazid-resistant strains in infected macrophages,^13^ it remains to be determined whether the collateral phenotypes in cell-wall synthesis of other DR strains can be targeted under host-relevant conditions. Combined, these results highlight how variations in cell-wall composition in DR strains alters antibiotic tolerance and an increased need to investigate the influence of antibiotic tolerance on treatment outcomes.

## Supplementary Material

dlae086_Supplementary_Data

## Supplementary data

Figures S1 to S4 and Tables S1 to S3 are available as Supplementary data at *JAC-AMR* Online.
