# Supplementary material for: Antibiotic resistance in Mycobacterium tuberculosis alters tolerance to cell wall-targeting inhibitors
Source: JAC Antimicrob Resist. 2024 Jun 4;6(3):dlae086. doi: 10.1093/jacamr/dlae086 (PMC11148391; doi:10.1093/jacamr/dlae086)
Supplement: dlae086_Supplementary_Data [file dlae086_supplementary_data.docx]

**Supplemental Material: Antibiotic resistance in *Mycobacterium* *tuberculosis* alters tolerance to cell wall-targeting inhibitors**

William J Jowsey, Gregory M Cook, Matthew B McNeil^*^.

Department of Microbiology and Immunology, University of Otago, Dunedin, New Zealand.

Corresponding author: matthew.mcneil@otago.ac.nz

**
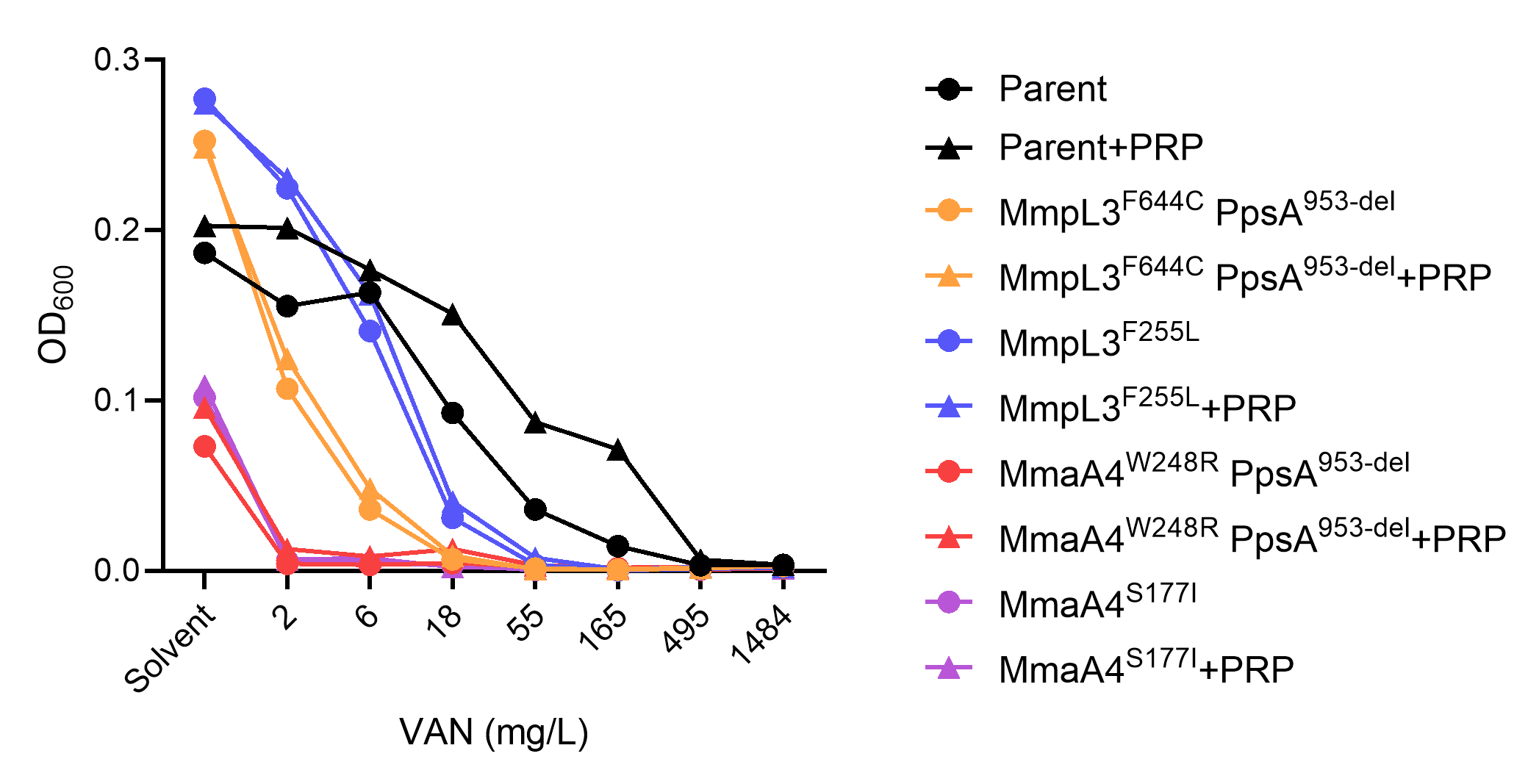
**

**Figure S1. OD_600_ values after challenge with VAN in the presence and absence of propionate**

Symbols show mean OD_600_ values from biological duplicate assays (each with two technical replicates). +PRP, supplemented with 0.1 mM propionate.

**
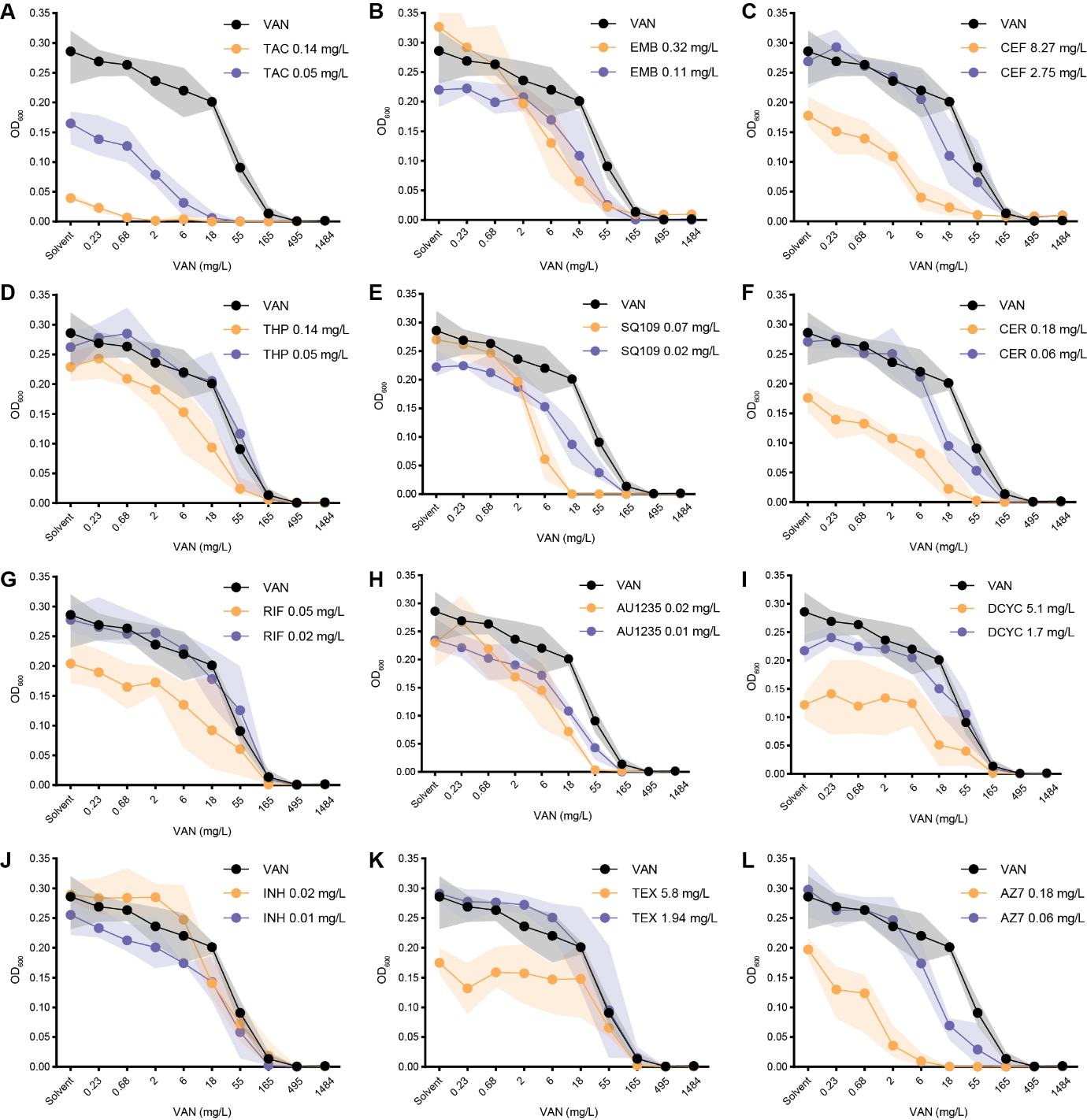
**

**Figure S2. OD_600_ values of the DS parent after challenge with VAN in combination of subinhibitory concentrations of cell wall-targeting antibiotics**

A-L) OD_600_ values from VAN challenges of the DS parent with subinhibitory concentrations of various cell wall-targeting antibiotics. Drug abbreviations: VAN, vancomycin, TAC, thioacetazone; EMB, ethambutol; CEF, ceftriaxone; THP, thiophene-2; CER, cerulenin; RIF, rifampicin; dCYC, d-cycloserine; INH, isoniazid; TEX, teixobactin; AZ7, AZ7371. Circles show mean OD_600_ values from biological duplicate assays (each with two technical replicates), and OD_600_ range is shown by background area. Orange and purple symbols refer to partner compound used at 0.33 and 0.11 x MIC of the DS-parent respectively. Drug concentrations rounded to 2 decimal places.

**
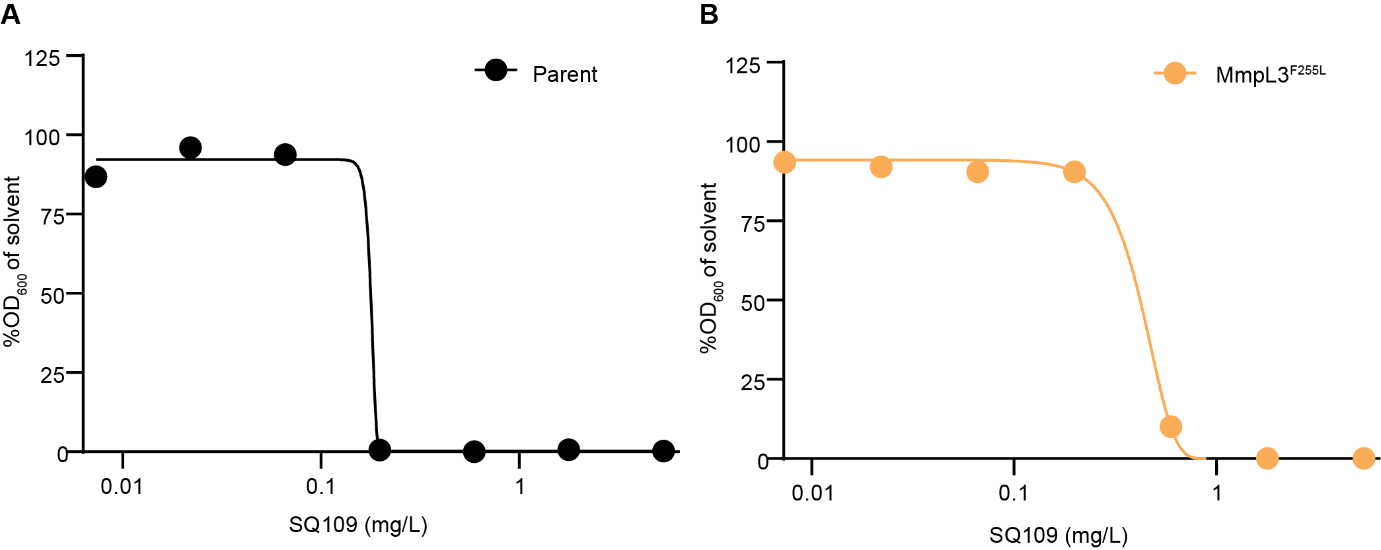
**

**Figure S3. MIC of the DS parent and MmpL3^F255L^ strain against SQ109**

A, B) MIC curves from SQ109 challenge against the DS parent and MmpL3^F255L^ respectively. Circles show mean %OD_600_ values (treatment/solvent OD_600_ × 100) from representative assays (each with two technical replicates).


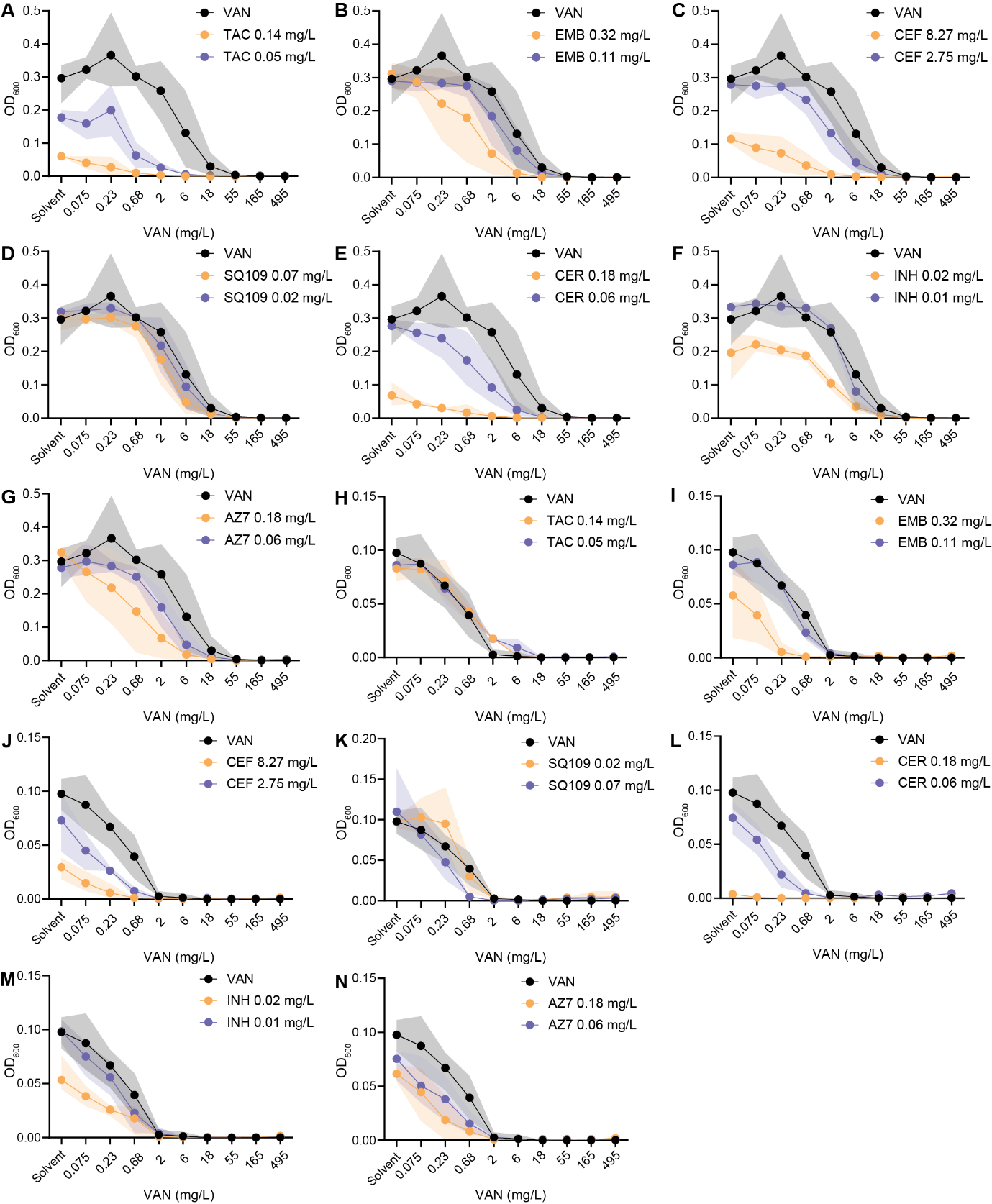


**Figure S4. OD_600_ values of the DR strains MmpL3^F255L^ and MmaA4^S177I^ after challenge with VAN in combination of subinhibitory concentrations of cell wall-targeting antibiotics**

A-G) and H-N) OD_600_ values of MmpL3^F255L^ and MmaA4^S177I^, respectively, following VAN challenges with subinhibitory concentrations of various cell wall-targeting antibiotics. Drug abbreviations: VAN, vancomycin, TAC, thioacetazone; EMB, ethambutol; CEF, ceftriaxone; CER, cerulenin; INH, isoniazid; AZ7, AZ7371. Circles show mean OD_600_ values from biological duplicate assays (each with two technical replicates), and OD_600_ range is shown by background area. Drug concentrations rounded to 2 decimal places.

**Table S1. Bacterial strains used in this study.**

| Strain | DNA change | PDIM gene mutations | Reference | Mutations call quality |
| --- | --- | --- | --- | --- |
| mc26206 | NA | NA | (1) | NA |
| RpoB^S450L^ | *rpoB*^C1349T^ | Wildtype | (2) | NA |
| FbiC^T707P^ | *fbiC*^A2119C^ | Wildtype | (2) | NA |
| KatG^L458QfsX27^ | *katG*^1373delCTTAA^ | Wildtype | (2) | NA |
| GyrA^D94Y^ | *gyrA*^G280T^ | Wildtype | (2) | NA |
| Ddn^K103KfsX58^ | *ddn*^307delA^ | Wildtype | (2) | NA |
| QcrB^M342V^ | *qcrB*^A1024G^ | Wildtype | (2) | NA |
| Pks13^T427A^ | *pks13*^T1279C^ | Wildtype | (2) | NA |
| DprE1^Y314H^ | *dprE1*^T940C^ | Wildtype | (2) | NA |
| *rrl*^A2269AT^ | *rrl*^A2269AT^ | Wildtype | (2) | NA |
| FusA1^S584A^ | *fusA*^T1750G^ | ppsB^T2867A^ | (2) | NA |
| AtpE^A63P^ | *atpE*^G187C^ | Wildtype | (2) | NA |
| RplC^C154R^ | *rplC*^T460C^ | Wildtype | (2) | NA |
| Gid^P75S^ | *gid*^G223A^ | fadD26^G128A^ | (2) | NA |
| *rrs*^C517T^ | *rrs*^C517T^ | fadD26^G128A^ | (2) | NA |
| QcrB^T313A^ | *qcrB*^A937G^ | Wildtype | (2) | NA |
| QcrB^T313A^ | *qcrB*^A937G^ | ppsA^2858delC^ | (2) | NA |
| Rv0678^65del^ | *rv0678*^193delG^ | Wildtype | (2) | NA |
| TlyA^K182N^ | *tlyA*^G546T^ | ppsA^2858delC^ | (2) | NA |
| MshA^G260DfsX18^ | *mshA*^778delG^ | Wildtype | (2) | NA |
| MshC^D253G^ | *mshC*^T758C^ | ppsC^T2330A^ | (2) | NA |
| MmpL3^F644C^ | *mmpL3*^A1931C^ | ppsC^2858delC^ | (2) | NA |
| MmpL3^F255L^ | *mmpL3*^A763G^ | Wildtype | (2) | NA |
| MmpL3^S591I^ | *mmpL3*^C1772A^ | ppsA^2858delC^ | (2) | NA |
| MmaA4^W248R^ | *mmaA4*^A742G^ | ppsA^2858delC^ | (2) | NA |
| MmaA4^S177I^ | *mmaA4*^C530A^ | ppsA^2858delC^ | (2) | NA |
| MmaA4^Y181X^ | *mmaA4*^543+T^ | Wildtype | (2) | NA |
| RpoB^Q1080R^ | *rpoB*^A3239G^ | Wildtype | (2) | NA |
| Isolate A | *yrbE1A*^429insCCCATCC^ | Wildtype | This study | 228.2 |
| Isolate B | *mam1D*^537delC^ | Wildtype | This study | 228.4 |
| Isolate C | *mam1D*^537delC^ | Wildtype | This study | 228.3 |

**Table S2. Antibiotic manufacturers and stock concentrations.**

| Antibiotic | Supplier | Cat. number | Stock concentration (mM) | Solvent |
| --- | --- | --- | --- | --- |
| AU1235 | Selleckchem | S0464 | 30 | DMSO |
| AZ7371 | Cayman | 19310 | 5 | DMSO |
| Ceftriaxone | Sigma-Aldrich | C5793 | 75 | DMSO |
| Cerulenin | Sigma-Aldrich | C2389 | 20 | DMSO |
| Clavulanate | Sigma-Aldrich | 33454 | 50 | Water |
| d-cycloserine |  |  | 100 | DMSO |
| Ethambutol | Sigma-Aldrich | E4630 | 50 | DMSO |
| Isoniazid | Sigma-Aldrich | I3377 | 50 | Water |
| Meropenem | Sigma-Aldrich | PHR1772 | 50 | DMSO |
| PBTZ-169 | Cayman Chemical | 22202 | 0.05 | DMSO |
| Penicillin | Sigma-Aldrich | 13752 | 180 | DMSO |
| Rifampicin | Sigma-Aldrich | R3501 | 2 | DMSO |
| SQ109 | Sigma-Aldrich | SML1309 | 2.5 | DMSO |
| Teixobactin |  |  | 8 | DMSO |
| Thioacetazone | Santa Cruz Biotechnology | sc-358574 | 20 | DMSO |
| Thiophene-2 | Sigma-Aldrich | SML1120 | 10 | DMSO |
| Vancomycin | Duchefa Biochemie | V0155.0005 | 67 or 33.6 | DMSO |

**Table S3. VAN MICs of the DS parent in combinations with subinhibitory concentrations of cell wall-targeting antibiotics.**

| Drug combination | ×MIC*^b^* | Concentration (mg/L) | VAN MIC (mg/L)*^a^* |
| --- | --- | --- | --- |
| VAN only | - | - | 148.4 |
| TAC | 0.33 | 0.14 | 1.3 |
| TAC | 0.11 | 0.05 | 10.3 |
| EMB | 0.33 | 0.32 | 37.0 |
| EMB | 0.11 | 0.11 | 70.6 |
| CEF | 0.33 | 8.27 | 8.8 |
| CEF | 0.11 | 2.75 | 106.6 |
| THP | 0.33 | 0.14 | 85.8 |
| THP | 0.11 | 0.05 | 184.6 |
| SQ109 | 0.33 | 0.07 | 9.6 |
| SQ109 | 0.11 | 0.02 | 92.5 |
| CER | 0.33 | 0.18 | 32.8 |
| CER | 0.11 | 0.06 | 77.5 |
| RIF | 0.33 | 0.05 | 149.5 |
| RIF | 0.11 | 0.02 | 193.2 |
| AU1235 | 0.33 | 0.02 | 61.0 |
| AU1235 | 0.11 | 0.01 | 102.5 |
| d-CYC | 0.33 | 5.1 | 110.2 |
| d-CYC | 0.11 | 1.7 | 208.4 |
| INH | 0.33 | 0.02 | 100.8 |
| INH | 0.11 | 0.01 | 135.3 |
| TEX | 0.33 | 5.8 | 76.8 |
| TEX | 0.11 | 1.94 | 130.4 |
| AZ7371 | 0.33 | 0.18 | 2.6 |
| AZ7371 | 0.11 | 0.06 | 35.2 |

*^a^*VAN MIC values were calculated from two biological replicate assays (each with two technical replicates) using nonlinear regression (curve fit) with the Gompertz equation. MIC values were rounded to one decimal place. *^b^*×MIC values were calculated from representative biological assays (each with two technical replicates) as described above (data generated during killing assays (Figure 1a); data not shown). Drug abbreviations: VAN, vancomycin, TAC, thioacetazone; EMB, ethambutol; CEF, ceftriaxone; THP, thiophene-2; CER, cerulenin; RIF, rifampicin; d-CYC, d-cycloserine; INH, isoniazid; TEX, teixobactin. Drug concentrations rounded to two decimal places.

**References**

1. Jain P, Hsu T, Arai M, *et al*. 2014. Specialized transduction designed for precise high-throughput unmarked deletions in *Mycobacterium tuberculosis*. mBio 2014. **5**:e01245-14. https://doi.org/10.1128/mbio.01245-14.

2. Waller NJE, Cheung C-Y, Cook GM, *et al*. The evolution of antibiotic resistance is associated with collateral drug phenotypes in *Mycobacterium tuberculosis*. Nat Commun 2023; **14**:1527. https://doi.org/10.1038/s41467-023-37184-7.
